# Clinical and genetic spectrum of dual rare genetic diseases revealed by whole-exome sequencing in 14 pediatric patients

**DOI:** 10.1186/s13023-026-04462-8

**Published:** 2026-09-21

**Authors:** Minjun Zhao, Fuwei Li, Xiangpeng Lu, Hong Zheng, Xilong Du

**Affiliations:** 1 School of Pediatrics, Henan University of Chinese Medicine, Zhengzhou, Henan China; 2 Department of Pediatrics, First Affiliated Hospital of Henan University of Traditional Chinese Medicine, Zhengzhou, Henan China; 3 Chigene (Beijing) Translational Medical Research Center Co.,Ltd., Beijing, China; 4 Beijing Quanpu Medical Laboratory Co., Ltd., E2, 3rd Floor, No. 88 Kechuang 6th Road, Beijing Yizhuang Biomedical Park, Beijing, 100176 China

**Keywords:** Dual molecular diagnosis, Whole-exome sequencing, Rare genetic diseases, Pediatric genetics

## Abstract

**Background:**

Dual molecular diagnoses, defined as the coexistence of pathogenic variants in two distinct disease-causing genes, challenge the traditional single-gene model of Mendelian inheritance. With the advent of whole-exome sequencing (WES), such complex genotypes are increasingly recognized.

**Objective:**

To investigate the clinical and genetic spectrum of dual rare genetic diseases in pediatric patients and to assess the diagnostic utility of WES in detecting diverse variant types.

**Methods:**

A retrospective analysis was conducted on 872 children with suspected rare genetic disorders who underwent proband or trio-based WES from January 2019 to February 2025. Variants were classified according to American College of Medical Genetics and Genomics(ACMG) guidelines. Dual diagnoses were confirmed when two independent pathogenic or likely pathogenic variants, each explaining part of the phenotype, were identified.

**Results:**

Among 872 patients, 370 (42.4%) received a molecular diagnosis, and 14 (3.8%) were confirmed with dual molecular diagnoses. Autosomal dominant (AD) disorders were most frequent (85.7%), de novo variants represented 57.14% (8 out of 14 cases). Six patients (42.9%) harbored combinations of single nucleotide variant (SNV) and other variant types, including exon deletions, paternal uniparental disomy (UPD15). Clinically, 14.3% of cases were phenotypically distinct, while 85.7% were phenotypically overlapping.

**Conclusion:**

Dual molecular diagnoses occur in approximately 3.8% of molecularly diagnosed patients and frequently involve de novo dominant variants. WES effectively detects multiple variant types (including SNV, CNV, exon deletions, UPD, and mosaicism) providing crucial insights for precise diagnosis, management, and genetic counseling in children with complex phenotypes.

## Introduction

Genetic diseases represent a diverse group of disorders caused by variations in the human genome, and are characterized by a wide variety of types, complex clinical manifestations, and strong genetic heterogeneity. According to statistics, more than 7,000 genetic diseases have been identified so far, affecting approximately 300 million people worldwide [1]. Traditionally, a single pathogenic variant in one gene was considered sufficient to explain a patient’s phenotype. However, with the rapid development of molecular diagnostic technologies, especially high-throughput sequencing technologies, more and more studies have revealed that some individuals harbor dual molecular diagnoses-pathogenic variants in two distinct genes, each responsible for separate or partially overlapping disorders [2–6]. Recent literature further validates this complexity, demonstrating that advanced genomic analyses are critical for resolving cases where blended phenotypes obscure single-gene diagnoses [7, 8]. This emerging concept has redefined the landscape of Mendelian genetics. Patients with dual diagnoses often present with atypical, blended, or unusually severe phenotypes, posing diagnostic challenges in clinical settings. These cases may arise from de novo mutations, compound inheritance across loci, or mosaic and structural variants [5, 6]. Previous large-scale studies estimate that dual or multilocus diagnoses occur in 1–7% of WES-tested patients, underscoring the clinical importance of considering multilocus pathogenicity [2–6].

This study retrospectively analyzed 872 pediatric patients with suspected genetic diseases who underwent WES, identifying 14 individuals with dual molecular diagnoses. We aimed to delineate their clinical and genetic spectrum, classify phenotypic presentations, and evaluate the diagnostic utility of WES, providing insights for precision diagnosis and genetic counseling in pediatric rare diseases.

## Materials and methods

### Study subjects

This retrospective study included 872 unrelated children with suspected genetic disorders evaluated between January 2019 and February 2025 at the Department of Pediatrics at the First Affiliated Hospital of Henan University of Chinese Medicine. All participants underwent proband or trio-based WES. Fourteen patients were confirmed to have dual molecular diagnoses. Inclusion required: (1) complex or atypical phenotype; (2) two independent pathogenic or likely pathogenic variants; and (3) each variant explaining part of the phenotype and corresponding to distinct OMIM-listed diseases. Written informed consent was obtained from guardians. Ethics approval was granted by the hospital’s ethics committee.

Clinical Data and Phenotyping: Clinical data were collected from electronic medical records, including demographic information, clinical symptoms, imaging, laboratory findings, and developmental assessments. Phenotypes were standardized using the Human Phenotype Ontology (HPO) and reviewed by two clinical geneticists.

### Whole exome sequencing

Peripheral blood samples were collected from patients and selected family members (primarily parents) for genomic DNA extraction. In this cohort, the diagnostic workflow prioritized WES as a first-tier screening tool for patients presenting with complex, multi-systemic, or atypical overlapping phenotypes (e.g., Patient 2’s concurrent neurodevelopmental and muscular symptoms), where a single-gene hypothesis might be insufficient. In specific cases with high clinical suspicion (e.g., Patient 11), WES and targeted MLPA were conducted concurrently to expedite diagnosis. WES was performed on 175 patients as single probands, while 697 patients underwent trio (proband and their parents) WES. Genomic exons were captured using the commercial kit IDT xGen Exome Research Panel v2.0, and high-throughput sequencing was performed on the BGI-T7 platform with a sequencing depth of ≥ 100×. The sequencing process was performed by Beijing Chigene Translational Medicine Research Center Co., Ltd.

### WES analysis and variant interpretation

Following quality control, raw sequencing data were aligned to the human reference genome (GRCh37/hg19) for variant detection, including single nucleotide variants (SNVs), small insertions and deletions, and copy number variants (CNVs) [9]. Variants were evaluated for pathogenicity and categorized as pathogenic, likely pathogenic, variants of unknown significance (VUS), likely benign, or benign, based on the standards and guidelines for genetic variant classification established by the American College of Medical Genetics and Genomics (ACMG) [10, 11], A dual molecular diagnosis requires both variants to be classified as either pathogenic or likely pathogenic, with each corresponding to a distinct genetic disease.

## Results

### Diagnostic yield

**Fig. 1:**
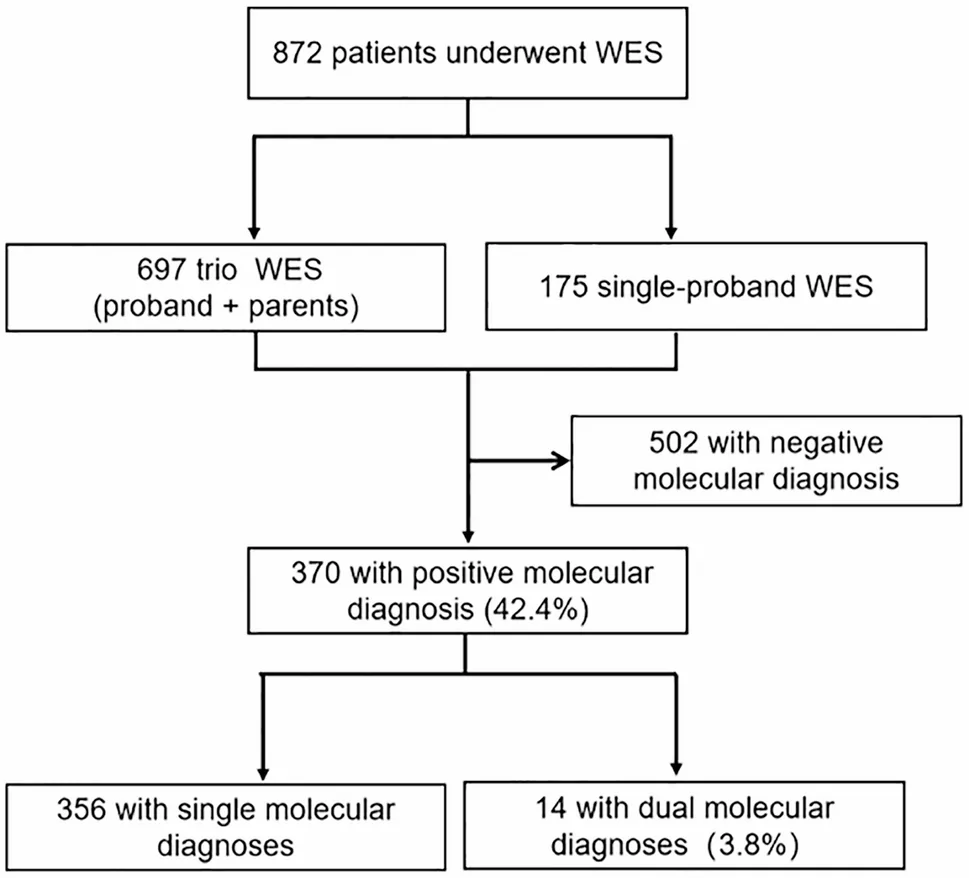
Flowchart of WES and molecular diagnosis outcomes. A total of 872 patients underwent WES (697 trio WES, 175 single-proband WES); 370 (42.4%) achieved positive molecular diagnosis, including 356 with single molecular diagnosis and 14 (3.8%) with dual molecular diagnosis; 502 patients had negative molecular diagnosis

**Fig. 2:**
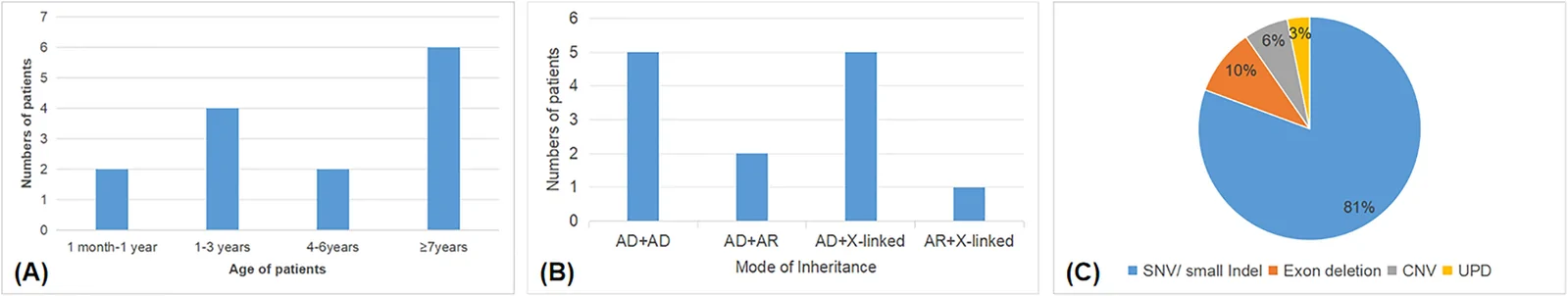
Composite plot of age, combined inheritance modes (dual diagnoses), and genetic variant type distributions. **A**: Age Distribution of Patients (≥ 7 years: *n* = 6; 1–3 years: *n* = 4; 1 month-1 year: *n* = 2; 4–6 years: *n* = 2). **B**: Distribution of Combined Inheritance Modes in Patients with Dual Molecular Diagnoses (AD + X-linked: 35.7%; AD + AD: 35.7%; other combinations: 28.6%). **C**: Proportional Distribution of Genetic Variant Types (SNVs/small Indels: 81%; exon deletions: 10%; CNVs: 6%; UPD: 3%)

Of 872 WES-tested patients, 370 (42.4%) received molecular diagnoses, including 14 (3.8%) with dual molecular diagnoses (Fig. 1). These 14 included nine males and five females (ages 2 months-15 years) (Table 1). Among the four age groups, the ≥ 7 years cohort had the largest number of patients (*n* = 6), followed by the 1–3 years group (*n* = 4). The 1 month–1 year and 4–6 years groups each included 2 patients. (Fig. 2A). None had parental consanguinity. The clinical phenotypes varied widely, affecting multiple systems, and the most frequent manifestations were neurological abnormalities (93%,13/14), developmental delay (79%,11/14), congenital malformations (71%,10/14), and intellectual disability (29% 4/14). In all cases, a single gene could not fully explain the clinical presentation.

**Table 1:** Clinical and molecular details of individuals with dual molecular diagnoses

Patient	Age	Gender	HPO	Gene	Transcript(NM)	chromosomal location	cDNA	AA Change	Zygosity	Inherited From	Associated Disease	Inheritance Mode	Variant Classification
1	8 years and 10 months	Male	Hypertelorism (HP:0000316), Joint contracture(HP:0034392),Ulnar claw(HP:0001178), Shield chest(HP:0000914),Café-au-lait spot (HP:0000957), Fever (HP:0001945), Abdominal distension (HP:0003270), Short stature (HP:0004322), Cough (HP:0012735), Night sweats (HP:0030166)	ABCD1	NM_000033	chrX:153,001,686–153,001,686	c.1202G > A	p.(Arg401Gln)	Hemizygous	maternal	Adrenoleukodystrophy; ALD (OMIM: 300100)	X-linked	Likely Pathogenic
				CHRNG	NM_005199	chr2:233406161–233,406,161	c.428 C > G	p.(Pro143Arg)	Heterozygous	maternal	Lethal Multiple Pterygium Syndrome; LMPS (OMIM: 253290)|Escobar Variant Multiple Pterygium Syndrome; EVMPS (OMIM: 265000)	AR	Pathogenic
						chr2:233408088–233,408,089	c.897_912dup	p.(Ile305Profs*98)	Heterozygous	paternal			Likely Pathogenic
2	8 years	Male	Intellectual disability (HP:0001249), Seizures (HP:0001250), Abnormal EEG (HP:0002353), Febrile seizures (HP:0002373), Genu valgum (HP:0002857), Myopathy (HP:0003198), Difficulty climbing stairs (HP:0003551), Muscular dystrophy (HP:0003560), Motor developmental delay (HP:0031936), Finger flexion (HP:0040019), Clinodactyly of the 5th finger(HP:0004209)	CSNK2A1	NM_177559	chr20:472926–472,926	c.593 A > G	p.Lys198Arg	Heterozygous	paternal	Okur-Chung Neurodevelopmental Syndrome (OMIM: 617062)	AD	Pathogenic
				DMD	NM_004006	chrX:32,519,871–32,563,451	loss(EXON:17–19)	44 K	Hemizygous	maternal	Becker Muscular Dystrophy (OMIM: 300376) |Duchenne Muscular Dystrophy/Progressive Pseudohypertrophic Muscular Dystrophy (OMIM: 310200)	X-linked	Likely Pathogenic
3	2 years and 6 months	Female	Abnormal stature (HP:0000002), Thin vermilion of the upper lip (HP:0000219), Long philtrum (HP:0000343), Telecanthus (HP:0000506), Jaundice (HP:0000952), Global developmental delay (HP:0001263), Prominent forehead (HP:0011220), Small hands (HP:0200055)	KIDINS220	NM_001348738	chr2:8943262–8,943,262	c.604-5T > G		Heterozygous	de novo	Spastic Paraplegia, Intellectual Disability, Nystagmus, and Obesity (OMIM: 617296)	AD	Likely Pathogenic
				ARID2	NM_152641	chr12:46123619–46,254,732	loss1(EXON:1–16)	131,114 bp	Heterozygous	de novo	Coffin-Siris Syndrome Type 6; CSS6 (OMIM: 617808)	AD	Pathogenic
4	4 years and 8 months	Male	Epicanthus (HP:0000286), Low anterior hairline (HP:0000294), Long philtrum (HP:0000343), Posteriorly rotated ears (HP:0000358), Protruding ears (HP:0000411), Heavy eyebrows (HP:0000574), Long palpebral fissure (HP:0000637), Autism/Autistic disorder (HP:0000717), Delayed speech and language development (HP:0000750), Intellectual disability (HP:0001249), Hypotonia (HP:0001252), Global developmental delay (HP:0001263), Neurogenic language disorder (HP:0002167), Short stature (HP:0004322), Widely spaced nipples (HP:0006610), Ectropion of lower eyelid (HP:0007651), Curvature of the middle phalanx of the 5th finger (HP:0009173), Short 5th finger (HP:0009237), Broad chin (HP:0012802), Irregular dentition (HP:0040079)	TBL1XR1	NM_024665	chr3:176756100–176,756,100	c.1047 + 1G > C		Heterozygous	de novo	Pierpont Syndrome (OMIM: 602342)|Mental Retardation, Autosomal Dominant 41; MRD41 (OMIM: 616944)	AD	Pathogenic
				PTCHD1	NM_173495	chrX:23,412,098–23,412,098	c.2463T > A	p.Cys821Ter	Hemizygous	de novo	X-linked Autism Susceptibility 4 (OMIM: 300830)	X-linked	Pathogenic
5	1 year and 1 month	Male	Renal enlargement (HP:0000105), Renal cysts (HP:0000107), High palate (HP:0000218), Abnormal forehead (HP:0000290), Delayed speech and language development (HP:0000750), Simian crease (HP:0000954), Global developmental delay (HP:0001263), Motor delay (HP:0001270), Decreased liver function (HP:0001410), Growth retardation (HP:0001510), Patent foramen ovale (HP:0001655), Gross motor developmental delay (HP:0002194), Abnormal electroencephalogram (HP:0002353), Language disorder (HP:0002357), Hyperphenylalaninemia (HP:0004923), Cerebral hypoplasia (HP:0006872), Lateral ventricular enlargement (HP:0006956), High anterior hairline (HP:0009890), Fine motor developmental delay (HP:0010862), Abnormal acetylcarnitine metabolism (HP:0012071), Hypoglycinemia (HP:0012277), Subarachnoid space widening (HP:0012704), Movement disorder (HP:0100660), Tongue thrusting habit (HP:0100703), Hypotyrosinemia (HP:0500133), Hypoprolinemia (HP:0500139), Hypoalaninemia (HP:0500154)	PKD1	NM_001009944	chr16:2161098–2,161,098	c.4070delT	p.Leu1357fsTer9	Heterozygous	paternal	Polycystic Kidney Disease Type 1 with or without Polycystic Liver Disease (OMIM: 173900)	AD	Likely Pathogenic
				NA	NA	chr15:1-102531392:upd1	Paternal uniparental disomy of chromosome 15	NA	NA		Angelman syndrome (OMIM:105830)		Pathogenic
6	17 years	Female	Dental abnormalities (HP:0000164), Myopia (HP:0000545), Intellectual disability (HP:0001249), Growth retardation (HP:0001510), Abnormal weight (HP:0004323), Short 5th finger (HP:0009237), Motor developmental delay (HP:0031936)	RERE	NM_012102	chr1:8416246–8,416,246	c.4400G > A	p.(Arg1467His)	Heterozygous	de novo	Neurodevelopmental Disorder with or without Abnormalities of the Brain, Eye, or Heart (OMIM: 616975)	AD	Likely Pathogenic
				NA	NA	chr7:72717592–74,148,323:loss1			Heterozygous	de novo		AD	Pathogenic
7	8 months	Male	Thin vermilion border (HP:0000233), Low anterior hairline (HP:0000294), Short philtrum (HP:0000322), Hypotonia (HP:0001252), Global developmental delay (HP:0001263), Muscle weakness (HP:0001324), Growth retardation (HP:0001510), Respiratory distress (HP:0002098), Short mandibular ramus (HP:0003778), Motor developmental delay (HP:0031936)	DVL1	NM_001330311	chr1:1273664–1,273,664	c.1492G > C	p.Gly498Arg	Mosaic	de novo	Autosomal Dominant Robinow Syndrome Type 2 (OMIM: 616331)	AD	Likely Pathogenic
				NKX2-1	NM_003317	chr14:36988301–36,988,302	c.263_c.264insA	p.Asn88fsTer321	Heterozygous	maternal	Benign Hereditary Chorea (OMIM: 118700)	AD	Likely Pathogenic
8	1 year and 4 months	Female	Global developmental delay (HP:0001263), Abnormal liver function (HP:0001410), Growth retardation (HP:0001510), Abnormal heart morphology (HP:0001627), Fever (HP:0001945), Pneumonia (HP:0002090), Hyperuricemia (HP:0002149), Lymphadenopathy (HP:0002716), Elevated liver transaminases (HP:0002910), Decreased plasma carnitine (HP:0003234), Tricuspid regurgitation (HP:0005180), Cough (HP:0012735), Motor developmental delay (HP:0031936), Pleural thickening (HP:0031944), Bronchial stenosis (HP:4000007)	SLC22A5	NM_003060	chr5:131705925–131,705,926	c.261_c.262insACCGGCTCGCC	p.Ile89fsTer45	Heterozygous	paternal	Primary Carnitine Deficiency (OMIM: 212140)	AR	Pathogenic
						chr5:131722736–131,722,736	c.844 C > T	p.Arg282Ter,276	Heterozygous	maternal			Pathogenic
				NA	NA	chr17:15133095–15,472,344:gain1			Heterozygous	de novo		AD	Pathogenic
9	14 years and 7 months	Female	Gonadal dysgenesis (HP:0000133), Delayed speech and language development (HP:0000750), Hypothyroidism (HP:0000821), Intellectual disability (HP:0001249), Global developmental delay (HP:0001263), Motor delay (HP:0001270), Excessive thyrotropin production (HP:0002925), Significant delay in bone age (HP:0003799), Short stature (HP:0004322), Female pubertal developmental disorder (HP:0008647), Abnormal uterine morphology (HP:0031105), Vitamin D deficiency (HP:0100512)	ASXL1	NM_015338	chr20:31019280–31,019,280	c.875 A > G	p.Asp292Gly	Heterozygous	de novo	Bohring-Opitz Syndrome/ “C-like” Syndrome (OMIM: 605039) |Myelodysplastic Syndrome Susceptibility; MDS (OMIM: 614286)	AD	Likely Pathogenic
				SOX10	NM_006941	chr22:38369948–38,369,948	c.957delG	p.Leu320Trpfs*37	Heterozygous	de novo	Peripheral Demyelinating Neuropathy, Central Dysmyelination, Waardenburg Syndrome, and Hirschsprung Disease/PCWH Syndrome (OMIM: 609136) |Waardenburg Syndrome Type 4 C (OMIM: 613266) |Waardenburg Syndrome Type 2E (OMIM: 611584)	AD	Pathogenic
10	1 year and 3 months	Male	Hydrocephalus (HP:0000238), Short philtrum (HP:0000322), Micrognathia (HP:0000347), Hypotonia (HP:0001252), Muscle weakness (HP:0001324), Weak cry (HP:0001612), Babinski sign (HP:0003487), Curved fingers (HP:0004095), Motor developmental delay (HP:0031936), Increased head circumference (HP:0040194), Cognitive impairment (HP:0100543)	MYH2	NM_017534	chr17:10430037–10,430,037	c.4066G > T	p.(Glu1356Ter,586)	Heterozygous	maternal	Congenital Myopathy Type 6 with Ophthalmoplegia; CMYP6 (OMIM: 605637)	AD	Likely Pathogenic
				MECP2	NM_001110792	chrX:153,296,354–153,296,354	c.961 C > T	p.(Arg321Trp)	Hemizygous	maternal	X-linked Autism Susceptibility 3; AUTSX3 (OMIM: 300496)| X-linked Intellectual Developmental Disorder, Lubs Type; MRXSL (OMIM: 300260)| Neonatal Severe Encephalopathy Due to MECP2 Mutation (OMIM: 300673)| X-linked Intellectual Developmental Disorder 13; MRXS13 (OMIM: 300055)| Rett Syndrome; RTT (OMIM: 312750)	X-linked	Pathogenic
11	11 years and 9 months	Male	Gait disturbance (HP:0001288), Abnormal calf muscles (HP:0001430), Foot abnormalities (HP:0001760), Achilles tendon contracture (HP:0001771), Hypertriglyceridemia (HP:0002155), Hyperhomocysteinemia (HP:0002160), Elevated liver transaminases (HP:0002910), Increased serum creatine phosphokinase (HP:0003236), Hypophosphatasia (HP:0003282), Increased serum pyruvate (HP:0003542), Muscular dystrophy (HP:0003560), Restricted ankle joint movement (HP:0010505), Decreased serum creatinine (HP:0012101), Unsteady gait (HP:0012651), Increased lactate dehydrogenase activity (HP:0025435), Increased circulating myoglobin concentration (HP:0033438)	RRAS2	NM_012250	chr11:14380347–14,380,348	c.70_78dup	p.(Gly24_Gly26dup)	Heterozygous	unknown	Noonan Syndrome Type 12; NS12 (OMIM: 618624)	AD	Pathogenic
				DMD	NM_004006	chrX:31,947,712–31,950,344	loss(EXON:46–47)		Hemizygous	de novo	Duchenne Muscular Dystrophy; DMD (OMIM: 310200)| Dilated Cardiomyopathy Type 3B; CMD3B (OMIM: 302045)| Becker Muscular Dystrophy; BMD (OMIM: 300376)	X-linked	Pathogenic
12	15 years and 4 months	Male	Irritability (HP:0000713), Dystonia (HP:0001332), Memory impairment (HP:0002354), Abnormal posture (HP:0002533), Involuntary movements (HP:0004305), Extrapyramidal movement disorder (HP:0007308), Fine motor developmental delay (HP:0010862), Cognitive impairment (HP:0100543)	GNAL	NM_182978	chr18:11752885–11,752,888	c.410_413delAACA	p.(Lys137Argfs*2)	Heterozygous	paternal	Dystonia Type 25; DYT25 (OMIM: 615073)	AD	Likely Pathogenic
				SGCE	NM_003919	chr7:94230140–94,230,140	c.855T > G	p.(Tyr285Ter,153)	Heterozygous	paternal	Myoclonus-Dystonia Syndrome Type 11; DYT11 (OMIM: 159900)	AD	Likely Pathogenic
13	2 months	Male	Cryptorchidism (HP:0000028), Hydrocele testis (HP:0000034), High palate (HP:0000218), Epicanthus (HP:0000286), Micrognathia (HP:0000347), Esotropia (HP:0000565), Thumb adduction (HP:0001181), Hypotonia (HP:0001252), Spastic paraplegia (HP:0001258), Global developmental delay (HP:0001263), Patent foramen ovale (HP:0001655), Gross motor developmental delay (HP:0002194), Subependymal cysts (HP:0002416), Neonatal hyperbilirubinemia (HP:0003265), Abdominal distension (HP:0003270), Abnormal weight (HP:0004323), Hyperreflexia of the knee tendon (HP:0007083), Bilateral cryptorchidism (HP:0008689), Fifth toe abnormality (HP:0010322), Prominent forehead (HP:0011220), Mongolian spot (HP:0011369), Cyanotic macule (HP:0033832)	NR0B1	NM_000475	chrX:30,322,932–30,322,932	c.1177G > T	p.(Gly393Cys)	Mosaic	de novo	Congenital Adrenal Hypoplasia; AHC (OMIM: 300200)| 46,XY Sex Reversal 2; SRXY2 (OMIM: 300018)	X-linked	Likely Pathogenic
				COL1A1	NM_000088	chr17:48263829–48,263,829	c.3854 A > G	p.(Asp1285Gly)	Heterozygous	de novo	Osteogenesis Imperfecta-Ehlers-Danlos Syndrome 1; OIEDS1 (OMIM: 619115)| Ehlers-Danlos Syndrome, Arthrochalasia Type 1; EDSARTH1 (OMIM: 130060)| Osteogenesis Imperfecta Type 2; OI2 (OMIM: 166210)| Osteogenesis Imperfecta Type 1; OI1 (OMIM: 166200)| Osteogenesis Imperfecta Type 3; OI3 (OMIM: 259420)| Caffey Disease; CAFYD (OMIM: 114000)| Osteoporosis (OMIM: 166710)| Osteogenesis Imperfecta Type 4; OI4 (OMIM: 166220)	AD	Pathogenic
14	4 years and 11 months	Female	Hypertelorism (HP:0000316), Café-au-lait spot (HP:0000957), Fever (HP:0001945), Abdominal distension (HP:0003270), Short stature (HP:0004322), Cough (HP:0012735), Night sweats (HP:0030166)	ACAN	NM_001369268	chr15:89382006–89,382,007	c.186_187insCTGTGACC	p.(Ala63Leufs*2)	Heterozygous	paternal	Spondyloepiphyseal Metaphyseal Dysplasia, Aggrecan Type; SEMDAG (OMIM: 612813)| Short Stature with Advanced Bone Age, with or without Early-Onset Osteoarthritis and/or Osteochondritis Dissecans; SSOAOD (OMIM: 165800)| Spondyloepiphyseal Dysplasia, Kimberley Type; SEDK (OMIM: 608361)	AD	Likely Pathogenic
				POLE	NM_006231	chr12:133226069–133,226,071	c.3832_3834delAAG	p.(Lys1278del)	Heterozygous	maternal	Intrauterine Growth Retardation, Metaphyseal Dysplasia, Adrenal Hypoplasia Congenita, Genital Anomalies, and Immunodeficiency; IMAGEI (OMIM: 618336)| Facial Dysmorphism, Immunodeficiency, Livedo, and Short Stature (FILS Syndrome); FILS (OMIM: 615139)	AR	Likely Pathogenic
						chr12:133220132–133,220,133	c.4306dup	p.(Arg1436Profs*14)	Heterozygous	paternal			Likely Pathogenic

NA: Not Applicable

### Inheritance patterns and variant types

Autosomal dominant diseases predominated (85.7%, 12/14), followed by X-linked (42.9%, 6/14) and autosomal recessive (21.4%) conditions. The most frequent combination was AD + X-linked, AD + AD(35.7%)(Fig. 2B). Among the 31 pathogenic variants, 13 were de novo, 18 inherited, and 1 unknown. Targeted follow-up of these families revealed clear phenotypic co-segregation in several cases; specifically, the fathers of Patient 2 and Patient 12 exhibited clinical features (intellectual disability and tremors, respectively) consistent with the identified autosomal dominant variants.

We analyzed the distribution of identified genetic variant types (as shown in Fig. 2C). The pie chart illustrates that single nucleotide variants (SNVs) and small insertions/deletions (small Indels) were the most predominant variant type, accounting for 81% of all detected alterations. Other variant categories contributed smaller proportions: exon deletions (10%), copy number variations (CNVs, 6%), and uniparental disomy (UPD, 3%). Six patients (42.9%) harbored combined SNVs plus other variant types (including CNVs, exon deletions, and UPD) — underscoring the importance of concurrent detection of multiple variant classes to enable comprehensive diagnostic evaluation. Exon deletions were found in three cases, and paternal UPD15 in one(patient5). Mosaic variants were identified in two patients: Patient 7 harbored a mosaic variant c.1492G > C (p.Gly498Arg) in the *DVL1* gene with a mosaicism rate of 20%, while Patient 13 carried a mosaic variant c.3854 A > G (p.Asp1285Gly) in the *NR0B1* gene with a mosaicism rate of 27%.

### Phenotype description

Analysis of the clinical and molecular characteristics of the 14 patients with dual molecular diagnoses revealed that 85.7% (12/14) exhibited partially overlapping phenotypic features between their two diagnosed conditions; the remaining 14.3% (2/14) presented distinct phenotypic profiles corresponding to each of the two disorders. **Patient 5** was a 13-month-old boy, presenting with developmental delay, unable to hold his head steadily, unable to sit, crawl or stand alone, and unable to say ‘dad’ or ‘mom’. Specialist examination: head circumference 46 cm, anterior fontanel closed, flat back of the head, high hairline at the frontal angle, sunken and narrow forehead, slightly high palatal arch, underdeveloped auricles, strabismus, simian crease, and high muscle tone. Cranial MRI showed: Widened subarachnoid space in the frontal and temporal lobes, mild dilation of lateral ventricles. Gesell result: severe developmental delay. Psychological behavior test: comprehensive developmental quotient DQ: 44.8 (moderate defect). Peabody Motor Development Scale conclusion: gross motor, fine motor and total motor development were significantly delayed. Color Doppler ultrasound: (1) Large bilateral kidneys with mild diffuse changes in parenchymal echo; (2) Bilateral renal cysts; (3) Mild separation of left renal pelvis (about 5 mm); (4) Patent foramen ovale (about 2.2 mm). Electroencephalogram: abnormal infant electroencephalogram, low-amplitude sharp waves in bilateral central and parietal regions during sleep, synchronous or asynchronous on both sides. Trio-WES revealed paternal uniparental disomy of chromosome 15 and a heterozygous variant [c.4070delT, p.Leu1357fsTer9] in the *PKD1* gene inherited from the father. Paternal uniparental disomy of chromosome 15 is associated with Angelman syndrome (OMIM:105830), which can lead to global developmental delay, language developmental delay, motor developmental delay, and facial malformations, consistent with the phenotype observed in this patient. The pathogenic variant identified in the *PKD1* gene may result in autosomal dominant polycystic kidney disease type 1, with or without polycystic liver (OMIM:173900), thereby explaining the renal cyst phenotype exhibited by the patient.

**Patient 3**, a 30-month-old girl, exhibited slow height growth. She was able to walk independently at 18 months and could say only ‘dad’ and ‘mom’. The patient presented with light-colored hair, a wide inner canthal distance, a long philtrum, slightly protruding eyeballs, downward-slanting outer canthi, a thick lower lip, a thin upper lip, small hands, sparse lower teeth, a slightly everted costal margin, and a protruding forehead with a central depression. Her comprehensive developmental quotient (DQ) was 63.8, indicating a mild defect. WES identified a heterozygous deletion variant spanning exons 1–16 in the *ARID2* gene, along with a c.604-5T > G variant in the *KIDINS220* gene, both of which were *de novo* mutations. Mutations in *ARID2* are associated with Coffin-Siris syndrome type 6 (OMIM: 617808) [12], which can lead to developmental delays and facial abnormalities in affected individuals. Conversely, mutations in *KIDINS220* are linked to spastic paraplegia, intellectual disability, nystagmus, and obesity (OMIM: 617296). According to the clinical evaluation at 30 months of age, Patient 3 exhibited developmental delay and significant motor impairment. However, nystagmus and obesity were absent, suggesting that these specific manifestations may be age-dependent. Additionally, reports have documented developmental delays and facial abnormalities in patients with *KIDINS220* mutations [13], indicating a phenotypic overlap associated with dual molecular diagnoses.

## Discussion

This study analyzed the WES results of 872 patients with suspected rare diseases, revealing that 14 cases (3.78%) exhibited dual molecular diagnoses. This proportion is consistent with the 1%-7% range reported in domestic and international studies [3, 4, 14], further confirming that dual rare genetic diseases are not merely sporadic anomalies but constitute a non-negligible genetic pattern among patients with complex phenotypes. This finding highlights the importance for clinicians to be vigilant for the potential of dual molecular diagnoses in patients with multi-system involvement and phenotypes not fully explained by a single disease. Family analyses can facilitate a more accurate distinction between *de novo* variants and inherited genetic variants by comparing the genotypes of the proband and parents, reducing the risk of missed diagnoses due to unknown variant origin [15].

In this study, autosomal dominant genetic diseases were identified as the most prevalent genetic pattern in dual diagnoses, accounting for 85.71% (12 out of 14 cases). Notably, *de novo* variants represented 57.14% (8 out of 14 cases), aligning with previous findings that “*de novo* variants are a significant cause of autosomal dominant genetic diseases in childhood” [16]. The high proportion of *de novo* variants explains why dual genetic diseases may manifest even in the absence of parental consanguinity, as germline mutations can arise spontaneously without being transmitted from parents. For instance, mosaic variants in *DVL1* and *NR0B1* were identified in two patients, exhibiting mutation rates of 20% and 27%, respectively. This suggests that mosaic variants may be an often-overlooked category in dual diagnoses, and the high-depth sequencing capabilities of WES provide essential technical support for their detection.

In terms of variant types, 42.86% (6/14) of dual diagnoses were attributed to the combination of SNVs and other variant types. Among these, three cases exhibited exon deletions, and one case was identified with paternal uniparental disomy of chromosome 15. This indicates that WES is capable of detecting not only point mutations but also structural variants, such as CNVs and uniparental disomy, which represents a significant advantage over traditional single-gene panels. For instance, patient 5 was found to have both paternal uniparental disomy of chromosome 15, which is associated with Angelman syndrome, and a frameshift variant in the *PKD1* gene. These findings respectively elucidate the phenotypes of developmental delay and renal cysts, thereby confirming the necessity of considering “multiple variants collaboratively explaining complex phenotypes.”

Phenotypic analysis revealed two categories among patients with dual diagnoses: ‘phenotype distinct’ and ‘phenotype overlapping.’ The Patient 5, exemplified by Angelman syndrome and autosomal dominant polycystic kidney disease type 1, is characterized by the absence of overlapping phenotypes. In contrast, Patient 3 includes conditions such as Coffin-Siris syndrome type 6 (*ARID2* variant) and spastic paraplegia (*KIDINS220* variant), both of which exhibit developmental delays and facial abnormalities. This overlap may arise from associations in gene function, such as participation in common biological pathways, and can complicate clinical diagnoses. WES provides a molecular basis for differentiating these two scenarios by simultaneously detecting multiple gene variants [17].

The limitations of this study include a small sample size (14 cases), which may constrain the generalizability of the conclusions. Furthermore, the functional implications of the variants have not been verified in vitro, and some phenotype-genotype associations require additional experimental validation. Future research should aim to expand the sample size and incorporate functional experiments and long-term follow-ups to further elucidate the interaction mechanisms of dual variants.

## Conclusion

Dual molecular diagnoses represent a meaningful subset of complex rare diseases rather than rare coincidences. WES enables comprehensive variant detection, improving diagnostic precision for pediatric patients with complex phenotypes. Early recognition of dual diagnoses supports personalized management, refined genetic counseling, and improved understanding of multilocus pathogenicity in rare disease genomics.

## Data Availability

All data generated or analysed during this study are included in this published article.
